# Multiple cerebral venous thrombosis as the first presentation of Behçet syndrome: case report and literature review

**DOI:** 10.1007/s11845-025-04236-4

**Published:** 2026-01-13

**Authors:** Asuman Orhan Varoglu, Bahadir Hosver, Basak Atalay

**Affiliations:** 1 Department of Neurology, Medical School, Istanbul Medeniyet University, Egitim Street. Fahrettin Kerim Gokay Street, Kadikoy, Istanbul, 34722 Turkey; 2 Department of Radiology, Medical School, Istanbul Medeniyet University, Istanbul, Turkey; 3 Department of Neurology, Medical Faculty, Istanbul Medeniyet University, Istanbul, Turkey

**Keywords:** Neurobehcet’s disease, Cerebral venous thrombosis, Behcet disease, Behçet’s syndrome (BS), Clinic

## Abstract

**Introduction:**

Cerebral venous thrombosis as the first manifestation of Behçet’s Syndrome (BS) is very rare. We describe a case of BS in which all four major cerebral venous sinuses, including the bilateral sigmoid sinuses, transverse sinuses, and superior sagittal sinuses, exhibited severe cerebral venous thrombosis.

**Case report:**

An 18-year-old man was brought to our hospital with a three-month-old, wide, throbbing headache in both hemispheres and double, blurred vision while looking to the right for the last week. Magnetic Resonance Imaging (MRI) showed extensive thrombosis involving the bilateral sigmoid sinuses, transverse sinuses, and superior sagittal sinus. Collagen tissue diseases and systemic thrombophilia were ruled out. The patient, who had a history of erythema nodosum and genital and oral aphthae, was evaluated as 3/6 (+) positive by the pathergy test, leading to the diagnosis of BS. Prednisolone 48 mg/day and azathioprine 2 × 50 mg/day were started, and the prednisolone dosage was lowered to be stopped in three to four months. For nine months, anticoagulant medicine was administered. Partial recanalization was seen on control MRI venography.

**Conclusions:**

It may be life-saving to consider Behçet syndrome as a differential diagnosis in patients with multiple cerebral venous thrombosis.

## Introduction

Behçet syndrome (BS) is a multisystem disease that is more common in male patients, affecting the recurrent oral and genital ulcers, as well as the ocular, skin, gastrointestinal, neurological, vascular, and locomotor systems. In nonselected big series, the prevalence of Neuro-behcet Syndrome (NBS) in BS ranges from 3% to 9%. There are two types of neurological diseases: parenchymal and nonparenchymal. Brainstem lesions, myelopathy, hemiparesis, dysphagia, epileptic seizures, and mental abnormalities are examples of parenchymal involvement; cerebral venous thrombosis, intracranial pressure syndrome, and acute meningeal syndrome are examples of nonparenchymal involvement. In Behçet’s disease, men are four times more likely than women to have neurological involvement. However, gender is not a factor in the severity of neurological involvement [1]. In around 5% of all instances of Neuro-Behçet syndrome, the initial manifestation of Behçet syndrome is neurological system involvement. There are extremely few examples in the literature where cerebral venous thrombosis is the primary presentation of Behçet’s disease (Table 1). However, parenchymal involvement is the most prevalent neurological system involvement in adult patients [2–9]. In this report, we report a case of Behçet’s Syndrome that presented with multiple cerebral venous thrombosis in the superior sagittal sinus, bilateral transverse sinuses, and sigmoid sinuses.

**Table 1:** Cases of cerebral venous thrombosis as the initial presentation of behcet syndrome

Auhtor	Gender	Age of disease (year)	Clinic Signs	Venous thrombosis	HLA B51	Pathergy test	Treatment	Prognosis
Gomes et al. (2022)	Female	12	Headeache	Right superficial Sylvian venous thrombosis	Positive	Negative	Acetazolamide, Colchicine, infliximab	Full recovery
Shah et al. (2024)	Female	28	Headeache, Blurred vision	Superior sagittal sinus thrombosis	NR (not reported)	NR (not reported)	Acetazolamide, Methylprednisolone, Cyclophosphamide, Tocilizumab	Partial recovery
Gupta et al. (2024)	Female	18	Headache, Encephalopathy, Left hemiparsia	Superior sagittal sinus thrombosis	Positive	Positive	Acetazolamide, Decompression, Antibiotic	Death
	Male	22	Binocular vision loss	Superior sagittal sinus thrombosis	Positive	Negative	Methylprednisolone, Colchicine	Full recovery
Ferreira et al. (2017)	Female	28	Binocular vision loss	Superior sagittal sinus thrombosis, Right sigmoid sinus, right internal jugular venous thrombosis	Negative	NR (not reported)	Methylprednisolone, Mycophenolate Mofetil, infliximab	Partial recovery
Powell et al. (2022)	Male	20	Headache, Nausea and vomiting, Binocular vision loss	Superior sagittal sinus thrombosis	NR (not reported)	NR (not reported)	Methylprednisolone, Azathioprine, Enoxaparin, Warfarin	Full recovery
Prasad & Verma (2023)	Male	32	Headache, Nausea and vomiting, Binocular vision loss	Superior sagittal sinus and Right transverse sinus thrombosis	Positive	Positive	Acetazolamide, Methylprednisolone, warfarin	Full recovery
Chaloupka et al. (2003)	Male	13	Headache, pholophobia	Superior sagittal sinus, Sigmoid sinus, and lateral left sinus thrombosis	NR (not reported)	NR (not reported)	Prednisone, Ciclosporine, Warfarin	Full recovery
Rahil et al. (2009)	Male	40	Headache, Vision loss	Superior sagittal, left transverse, and left sigmoid sinus thrombosis	NR (not reported)	NR (not reported)	Methylprednisolone, Cyclophosphamide, Azathioprine, Warfarin	Full recovery

## Case report

**Fig. 1:**
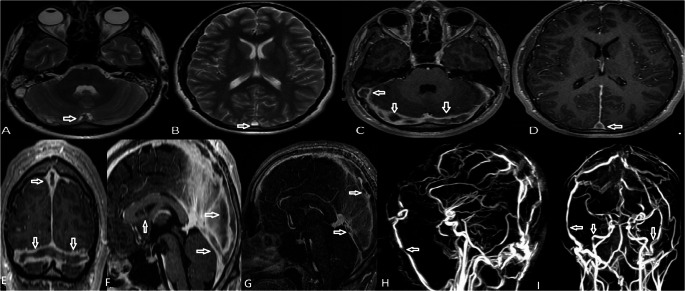
(**A**, **B**) Axial T2-weighted images demonstrate loss of the normal flow void in bilateral sigmoid sinuses, transverse sinuses, and superior sagittal sinus, consistent with thrombosis. (**C**, **D**) Postcontrast 3D T1-weighted axial images show a lack of enhancement in the same venous structures, confirming extensive thrombosis involving the bilateral sigmoid sinuses, transverse sinuses, and superior sagittal sinus. (**E**-**G**) Postcontrast 3D T1-weighted axial and sagittal images demonstrate the absence of normal enhancement in the bilateral sigmoid sinuses, transverse sinuses, sinus rectus, and superior sagittal sinus, consistent with extensive cerebral venous sinus thrombosis. **(H**,** I)** MR venography MIP images demonstrate extensive cerebral venous sinus thrombosis. Due to the absence of flow in the dural sinuses caused by thrombosis, the venous system is not visualized, and unintended visualization of the arterial system is observed

An 18-year-old male patient was brought to our hospital complaining of double and blurred vision while looking to the right, which he had been experiencing for the last week, as well as a broad, throbbing headache in both hemispheres for around three months. In his medical history, no previous illnesses, no chronic medication use, no smoking, and no family history of thrombosis. Neurological examination of the patient revealed 6th cranial palsy in the right eye. A fundus examination revealed bilateral papilledema of grade I–II. Bilateral visual fields showed peripheral contrastic narrowing. Visual acuity: 14/18, decreased on the right. MRI showed extensive thrombosis involving the bilateral sigmoid sinuses, transverse sinuses, and superior sagittal sinus (Fig. 1A-D). Lumbar puncture investigation showed that the patient’s cerebrospinal fluid (CSF) pressure was measured at 20 cmH_2_O. Cerebrospinal fluid biochemistry was normal, and the meningitis panel was negative. Multiple cerebral venous thromboses were seen on MR venography (Fig. 1G-I). The patient was started on anticoagulant therapy.

**Fig. 2:**
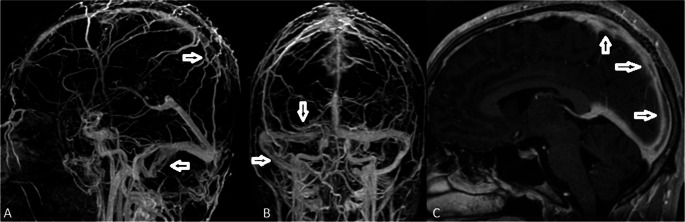
(**A**–**C**) Maximum intensity projection (MIP) MR venography images obtained one month after treatment demonstrate partial recanalization of the right transverse and superior sagittal sinuses, indicated by open white arrows

Laboratory and genetic results (protein C, protein S, antithrombin III, factor V Leiden, prothrombin G20210A, homocysteine, antiphospholipid antibodies) were normal. According to the ICBD 2014 criteria (10), Behçet syndrome was diagnosed after the patient, who had a history of genital and oral aphthous lesions as well as erythema nodosum, was assessed as 3/6 (+) positive by the pathergy test. HLA B-51 was negative. We excluded idiopathic intracranial hypertension, primary thrombophilias, systemic lupus erythematosus, other vasculitides, and infectious diseases. No thrombus was seen when thrombus scanning was done in the other areas. He began taking azathioprine twice a day at 50 mg and prednisolone 48 mg as part of an immunosuppressive regimen. Due to an improvement in his present symptoms, we discharged him. The patient received immunosuppressive and anticoagulant medication while being followed in the outpatient clinic, and an MRI venography was performed about a month later. Control MRI venography showed partial recanalization (Fig. 2A- C).

The patient was followed in our outpatient clinic at 1, 3, 6, and 12 months following his discharge. His headache and diplopia disappeared after about a month and three months, respectively. The patient’s neurological and ophthalmological evaluations were determined to be normal. Anticoagulant medication was stopped after approximately nine months, and the patient received immunosuppressive therapy without complications [10].

## Discussion

BS is an idiopathic, chronic, relapsing, multisystem vascular-inflammatory disorder. The neurological involvement, known as Neuro-Behcet syndrome, typically manifests years after BS is diagnosed, and it occurs in 5–10% of all cases of BS. There might be two different types of neurological involvement of BS [1]. The first type is parenchymal involvement (intra-axial), the most common type, which mostly presents as subacute brainstem syndrome and hemiparesis. The second type (extra-axial) has a better prognosis and presents as cerebral venous sinus thrombosis. It is quite uncommon for a patient to have both intra-axial and extra-axial types of NBS [1]. The authors claim that the development of the intra-axial type and the extra-axial type of NBS has different pathomechanisms. In BS, the pathophysiological factors behind the development of thrombosis remain unknown. The authors acknowledge the possibility that it is an immune-mediated endothelium disease. In 2025, the authors claimed that patients with BS who had a Factor V Leiden mutation had a 2.58-fold higher risk of thrombosis. Additionally, patients with BS and thrombosis had considerably higher levels of homocysteine and factor VIII. When compared to BS patients with thrombosis, non-BS patients with thrombosis had considerably greater rates of JAK-2 mutation, activated protein C resistance, and tissue plasminogen activator (tPA) levels, as well as tPA activity [1]. In every case that has been reported to date, including ours, superior sagittal sinus thrombosis has been noted [4–8]. Only the Gomes instance, the youngest case ever recorded, had a decreased caliber of the sigmoid, transverse, and superior sagittal sinuses rather than thrombosis [9]. Among the few BS patients whose first presentation sign is cerebral venous thrombosis, we find that all of them were extremely young and experienced multiple venous thrombosis, which involves more than one venous sinus.

The authors have stated the co-occurrence of coagulation factor XIIIA and HLA-B51 on chromosome 6p as a possible contributor to the pathophysiology of thrombosis in BS [11]. It is also claimed that the main treatment for a thrombotic event in BS should be immunosuppressive therapy rather than anticoagulants [12].

Corticosteroids (CS) are very useful in controlling Behçet’s disease during attacks and preventing further attacks. Adding it to the treatment plan until long-term immunosuppression is achieved is also important. For a patient with Behçet’s disease, long-term usage of immunosuppressants (such as azathioprine) is crucial to control of disease. Therefore, corticosteroids, azathioprine, and anticoagulants were administered to our patient. The patient was followed under immunosuppressive treatment without any problems following the cessation of anticoagulant therapy [13]. Given that in patients with cerebral venous thrombosis, which is the first clinical manifestation of Behçet’s syndrome, it often manifests with multivascular involvement and at a very young age, we believe that inflammatory processes may play a more prominent role in the development of cerebral venous thrombosis than thrombotic processes. To our knowledge, cerebral venous thrombosis in the follow-up period in cases with systemic Behçet Syndrome has not been shown to involve multiple vascular areas. In our case, the patient did not receive any anti-inflammatory medication, and the cerebral venous thrombosis affected several vascular areas in both hemispheres. After the diagnosis, we received anti-inflammatory and anticoagulant therapy, which resulted in a complete clinical cure. Anti-inflammatory medication appears to be more successful than anticoagulant treatment in cases of cerebral venous thrombosis occurring in Behçet’s Syndrome.

## Conclusion

Behçet’s Syndrome should be considered when cerebral venous thrombosis occurs in young individuals with involvement of multiple vascular branches. Also, it should not be forgotten that anti-inflammatory treatment is life-saving in patients with cerebral venous thrombosis of Behçet’s Syndrome.
